# Supplementary figures and images for: Comprehensive financial health assessment using Advanced machine learning techniques: Evidence based on private companies listed on ChiNext
Source: PLoS One. 2024 Dec 12;19(12):e0314966. doi: 10.1371/journal.pone.0314966 (PMC11637307; doi:10.1371/journal.pone.0314966)

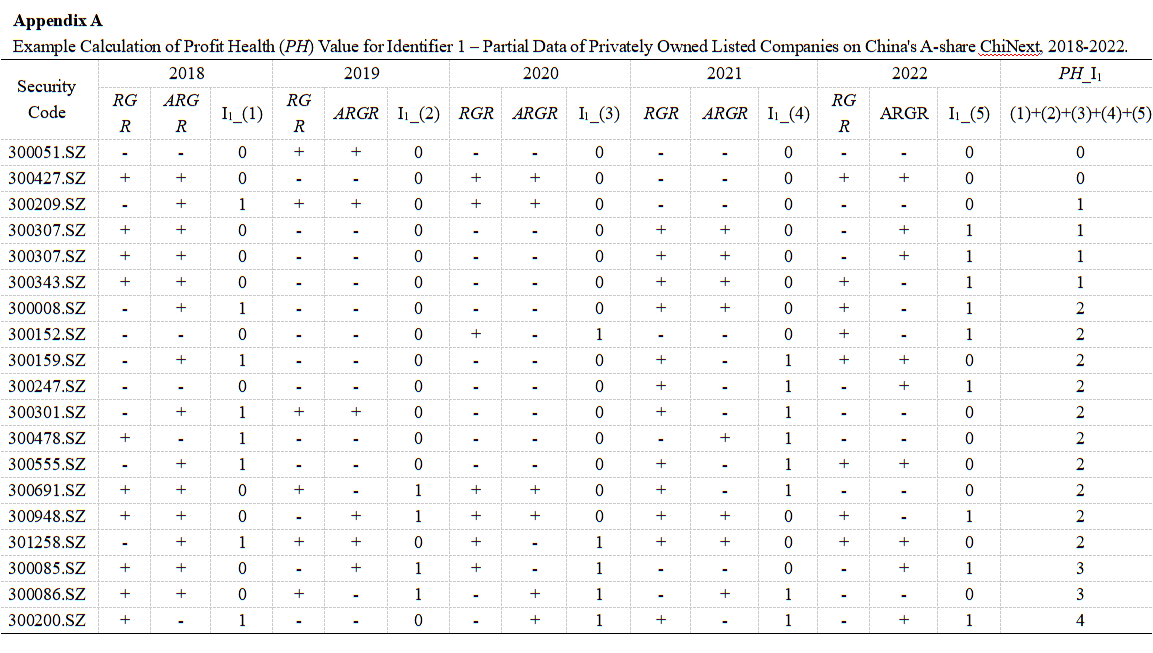

Supplement: S1 Appendix — (TIF) [file pone.0314966.s001.tif]
