# Supplementary material for: Comprehensive financial health assessment using Advanced machine learning techniques: Evidence based on private companies listed on ChiNext
Source: PLoS One. 2024 Dec 12;19(12):e0314966. doi: 10.1371/journal.pone.0314966 (PMC11637307; doi:10.1371/journal.pone.0314966)
Supplement: Attachment — Submitted filename: Response to reviewers comment.docx [file pone.0314966.s004.docx]

Respected reviewers:

We express our heartfelt gratitude to you for once again sparing precious time from your hectic schedule to provide valuable comments on this paper. Upon receiving your review remarks, our team has once again carried out comprehensive and painstaking revisions to the paper. The following are our detailed responses to each of your revision suggestions.

Abstract
Note: be more specific and identify the area and/or time period you are focusing on
R: Done

Note: clearly identify the aim of your study/research
R: See the SMART requirements and modify the aim based on them.

Reply: Based on your suggestion, the summary has been revised as follows:

**Abstract:** This study develops a specific and measurable framework for assessing the financial health (FH) of privately-owned companies listed on ChiNext, aimed at identifying financially sound enterprises and helping investors avoid losses caused by financial fraud or earnings management. The research proposes and tests four hypotheses related to key financial indicators and one overarching hypothesis regarding the model’s performance. Using gradient boosting machines and random forests, the model achieves high accuracy and robustness against overfitting through iterative learning. The framework incorporates four pairs of financial indicators and two non-financial indicators into four classifiers, significantly outperforming the Altman Z-score model in predicting financial soundness. Among 75 private companies with special treatment by the Securities Regulatory Commission in Shanghai and Shenzhen in 2022, 72 were correctly identified as sub-healthy or unhealthy, achieving an accuracy rate of 96%. This study demonstrates time-bound practical value by validating the model with 2022 data and highlights its relevance for cross-market applications. The results provide achievable solutions for enterprise managers and policymakers in financial decision-making and risk management.

Note: more focus on the methodology description and add other important information (be more specific)
R: Done.

Note: findings are too general (model can effectively differentiate....; validity of the proposed framework in practical applications is verified ...)
R: Done.

Note: explain the acronym "ST"
R: Done. I personally prefer to not use this acronym in this section (it looks weird that you have a note there)

Reply: Based on your feedback, I have removed this acronym and replaced it with the phrase 'with special treatment by the Securities Regulatory Commission'

Introduction
Note: what is a research question you are answering on? what is a research gap that will be solved by your research?
R: Partially done.

Reply: According to your feedback, relevant research questions and descriptions of research gaps have been added to the Introduction, as follows:

This study addresses the research question: How can machine learning models enhance the effectiveness of profit health assessment by integrating both financial and non-financial indicators? While existing research primarily focuses on financial fraud detection and earnings manipulation, there is a gap in providing comprehensive profit health assessments. The absence of methods combining financial and non-financial indicators limits the ability of investors and stakeholders to quickly and accurately identify companies with healthy financial conditions. To address this gap, this study builds on theories of earnings management, financial distress, and signal transmission to construct a profit health assessment framework. This model aims to overcome the limitations of traditional financial ratios by using a hybrid approach that integrates financial and non-financial data.

Note: add more relevant sources (3 is not enough)
R: Done.

Note: clearly identify the aim of your study/research
R: See the SMART requirements and modify the aim based on them.

Reply: According to your feedback, the aim has been revised as follows:

To address this gap, this study builds on theories of earnings management, financial distress, and signal transmission to construct a profit health assessment framework. This model aims to overcome the limitations of traditional financial ratios by using a hybrid approach that integrates financial and non-financial data. Furthermore, this study endeavours to combine this model with the Z-score model to enhance the effective identification of and ability to classify corporate financial health status [7], which offers an important assessment tool for financial conditions and risks to corporate stakeholders.

Note: highlight the structure of your manuscript
R: Check other manuscript and prepare appropriate paragraph with the structure description.

Reply: According to your feedback, the structure description has been added in the introduction as follows:

The article introduces, analyzes empirically, and applies the financial health model through the following chapters:

- **Introduction**: Establishes the context, research questions, and research gap.
- **Literature Review**: Summarizes key theories and previous studies.
- **Theoretical Framework and Hypotheses**: Proposes research hypotheses and conceptual models.
- **Methodology**: Details the research methods and data collection strategies.
- **Empirical Research**: Variable definition and model construction
- **Empirical Results and Robustness Test**: Analyzes the empirical results and ensures their stability.
- **Aplication and Expansion**: Application of profit health evaluation and extend to financial assessment
- **Conclusion** : Highlights the contributions and practical implications of the study.

Theoretical Framework and Research Hypotheses
Note: H1, H2 - there are too many variables in one hypothesis and it this was the hypotheses cannot be evaluated correctly (focus on 2 variables for each hypotheses)
R: Explained.

Note: H1, H2 - how it will be verified? ("...and inconsistent trends in these areas may signal unhealthy profits")
R: Explained.

Note: H3, H4 - how it will be verified? ("When inconsistent trends are exhibited in these two areas, it may indicate unhealthy profit signals.")
R: Explained.

Note: each hypothesis should be explained/described using another research results (why you are expecting the relationship between these indicators?) The current description represents your own point of view that should be support by other authors/researchers.
R: Explained.

Note: add information about the number of hypotheses in the abstract and the introduction
R: Done.

Note: the overarching model hypothesis H - what is a purpose/idea of its formulating?
R: Explained.

Methodology
Note: a separate section focusing on the methodology is missing; some information are mentioned in the section "Theoretical Framework and Research Hypotheses", other ones in the section "Empirical Research); from my point of view, the separate section should be created with all information required
R: Done.

Empirical Research
Note: Sample Selection and Data Collection - identify the number of subjects that are evaluated, describe them
R: Done.

Note: Variable definitions - identify the units for each variable + add information about the time period evaluated
R: Done.

Note: Descriptive statistics - the figures should be prepared with better quality, why not all variables are described?
R: Explained.

- Correlation Analysis - the CA should be included in the methodology
R: Explained.

Empirical Results Analysis and Robustness Test
Note: highlight the structure of this section on its beginning
R: Check other manuscript and prepare appropriate paragraph with the structure description.

Reply: According to your feedback, the structure description has been added in this section on its beginning as follows:

This section primarily pertains to the examination of two categories of models. Firstly, the gradient boosting machine algorithm is employed to test the overall model, thereby validating the combined impact of nine financial and non-financial indicators on profit health (*PH*). Secondly, the random forest algorithm is utilized to test four sub-models, with the aim of verifying the influence of a single detector on profit health (*PH*).

Note: Table 3 - move it as an annex
R: Done.

Theoretical Application
Note: the title should be changed (the content is different)
R: Done.

Conclusion
Note: add information about the limitations of the research presented
R: Done.

Formal
Note: check the journal template
R: Done.
